# Evaluation of eliciting activity of peptidil prolyl cys/trans isomerase from *Pseudonomas fluorescens* encapsulated in sodium alginate regarding plant resistance to viral and fungal pahogens

**DOI:** 10.3934/microbiol.2018.1.192

**Published:** 2018-03-12

**Authors:** Sophya B. Popletaeva, Natalia V. Statsyuk, Tatiana M. Voinova, Lenara R. Arslanova, Anton L. Zernov, Anton P. Bonartsev, Garina A. Bonartseva, Vitaly G. Dzhavakhiya

**Affiliations:** 1 Department of Molecular Biology, All-Russian Research Institute of Phytopathology, Bolshie Vyazemy, Moscow region, 143050, Russia; 2 Department of Potato and Vegetable Diseases, All-Russian Research Institute of Phytopathology, Bolshie Vyazemy, Moscow region, 143050, Russia; 3 Federal Research Centre “Fundamentals of Biotechnology” of the Russian Academy of Sciences, Moscow, 119071, Russia

**Keywords:** FKBP-type peptidyl prolyl cis-trans isomerase, PPIase, sodium alginate, encapsulation, protein elicitors, induced resistance of plants, plant pathogens, tobacco mosaic virus, *Alternaria longipes*, *Stagonospora nodorum*

## Abstract

Use of chemical pesticides poses a threat for environment and human health, so green technologies of crop protection are of high demand. Some microbial proteins able to activate plant defense mechanisms and prevent the development of resistance in plant pathogens, may be good alternative to chemicals, but practical use of such elicitors is limited due to need to protect them against adverse environment prior their delivery to target receptors of plant cells. In this study we examined a possibility to encapsulate heat resistant FKBP-type peptidyl prolyl cis-trans isomerase (PPIase) from *Pseudomonas fluorescens*, which possesses a significant eliciting activity in relation to a range of plant pathogens, in sodium alginate microparticles and evaluated the stability of resulted complex under long-term UV irradiation and in the presence of proteinase K, as well as its eliciting activity in three different “plant-pathogen” models comparing to that of free PPIase. The obtained PPIase-containing microparticles consisted of 70% of sodium alginate, 20% of bovine serum albumin, and 10% of PPIase. In contrast to free PPIase, which lost its eliciting properties after 8-h UV treatment, encapsulated PPIase kept its eliciting ability unchanged; after being exposed to proteinase K, its eliciting ability twice exceeded that of free PPIase. Using “tobacco-TMV”, “tobacco-*Alternaria longipes*”, and “wheat-*Stagonospora nodorum*” model systems, we showed that encapsulation process did not influence on the eliciting activity of PPIase. In the case of the “wheat-*S. nodorum*” model system, we also observed a significant eliciting activity of alginate-albumin complex and almost doubled activity of encapsulated PPIase as compared to the free PPIase. As far as we know, this is the first observation of a synergistic interaction between alginate and other compound possessing any bioactive properties. The results of the study show some prospects for a PPIase use in agriculture.

## 1. Introduction

Contemporary intensive agriculture provides for active use of various chemical preparations to protect crops against diseases, pests, and weeds. As a result, pesticides are accumulated in the soil and agricultural products that poses a serious threat to both environment and human health. Therefore, alternative environmentally safe technologies of crop protection are of high demand including those providing for the use of compounds of biological origin. A traditional trend in this field of studies is the development of crop protection tools based on antagonists of plant pathogenic microorganisms or metabolites produced by such antagonists. However, modern science reached a level that made it possible to develop a new strategy of plant protection based on the activation of natural plant defense mechanisms by so-called biogenic elicitors and resulting in a development of induced plant resistance to pathogens. This strategy deserves special attention, since it is environmentally safe and provides a long-term systemic effect in relation to a wide range of pathogens; moreover, the lack of any direct effect on plant pathogens prevents the appearance of pathogen resistance to the used compound [1],[2].

Elicitors represent exogenous or endogenous compounds able to activate various signal transduction pathways in plant cells triggering induced resistance and consequently activation of plant immunity to biotic and abiotic stresses. To date, a wide range of biogenic elicitors has been described including intact bacterial spores, microbial cell wall fragments, poly- and oligosaccharides, glycoproteins, lipids, organic acids, and also proteins and peptides. The last group includes some protein of microbial origin, such as harpins, flagellin, EF-Tu, cold shock proteins, etc. [3]–[8]. Authors of this study revealed the elicitor activity of a heat-resistant protein isolated from *Pseudomonas fluorescens* and belonging to the family of FKBP-type peptidyl prolyl cis-trans isomerases [9],[10]. This microbial enzyme was called PPIase; the further studies allowed us to determine its primary structure and the corresponding encoding nucleotide sequence and demonstrated the ability of this protein to elicit resistance to some fungal and viral pathogens in wheat, potato, rice, cabbage, and tobacco plants. The revealed protective effect was provided by the spraying of PPIase on the surface of seeds, tubers, and vegetating plants.

Developing biopreparations based on plant resistance inductors, one should take into account these compounds should interact with receptors of plant cells [11]–[14]. According to some authors, peptide and protein elicitors, characterized by a stronger specificity of interaction with target receptors, should provide better protection efficiency than organic compounds, which have more simple structure [15],[16]. However, the existing commercial preparations of such type are based mainly on bacterial strains, chitosan, organic acids, and other organic compounds [14],[17]. Such situation is explained by two main reasons. First, use of protein elicitors requires inexpensive technology of their large-scale production. Second, classical schemes of protective treatments provide for the spraying of used preparations on plants or seed material. In this case, protein, which remains on a leaf or seed surface or probably penetrates into plant tissues, is exposed to various plant proteases; in addition, it may be destructed by solar ultraviolet (UV) radiation [18]. All this reduces the efficiency of protein elicitors and limits the possibility of their practical use.

One of the possible ways to solve this problem is the use of protein elicitors in a complex with biopolymers able to protect them against adverse external factors and facilitate their interaction with plant cell receptors while keeping their biological activity [19],[20]. The range of suitable biopolymers includes polyethylene glycol, zwitterionic polymers and some other compounds. In recent years, protected delivery of various bioactive compounds to target receptors is provided mainly by encapsulation of these compounds using various polycations, such as sodium alginate (ALG) [21],[22]. This compound is of special interest due to its own elicitor activity [23],[24] and the possibility to use it as a biodegradable basis for encapsulation of various compounds including proteins and beneficial bacteria [25],[26]. According to existing publications, ALG also possesses some protective activity against animal pathogens via the stimulation of immune responses similar to plant defense reactions [27].

Though complexation of bioactive compounds with biopolymers is widely used in medicine for drug protection and delivery [21],[28], the use of this approach in agriculture is limited mainly by encapsulation of beneficial soil bacteria [29],[30]. Therefore, the problem of development of “protein elicitor-biopolymer” complexes characterized by improved resistance to adverse environmental factors still remains poorly studied.

The purpose of this study was the obtaining of a complex of PPIase with ALG, the assessment of the effect of UV radiation and protease treatment on the eliciting activity of PPIase alone and within such complex, as well as the study of the eliciting ability of the obtained complex in relation to some fungal and viral pathogens.

## 2. Materials and methods

### 2.1. PPIase production and isolation

PPIase was produced using a recombinant overproducing *E. coli* BL21 (DE3) strain developed during our earlier studies [31]. Strain cultivation was carried out according to the earlier described procedures [32]. Stock culture (3 mL) was added into flasks containing 100 mL of vegetative YT medium supplemented with ampicillin (1 µg/mL). Seed culture was grown on an Infors RC-TK thermo-shaker (Infors HT, Denmark; 5-cm orbit) for 20–22 h at 37 °C and 250 rpm and then transferred into 2-L fermenter containing 1.6 L of TV fermentation medium. To prepare 1 L of TV fermentation medium, 12 g of Bacto tryptone (Difco), 24 g of yeast extract, and 4 mL of glycerol were dissolved in 900 mL of distilled water, then sterilized, cooled to 60 °C, and supplemented with 100 mL of sterile 0.1 M phosphate buffer (pH 7.2) containing ampicillin (10 µg/mL). During fermentation, pO_2_ was maintained at the level of 0.5 L/L/min. To stimulate protein synthesis, 0.05 M isopropyl β-D-1-thiogalactopyranoside (IPTG) was added after 4 h to a final concentration of 1 mL/L. Fermentation was carried out for 20–22 h at 37 °C.

PPIase isolation was performed according to the earlier described procedure [32]. Culture broth was centrifuged for 30 min at 4,000 g. Precipitated cells were resuspended in 50 mM Tris-HCL buffer (pH 8.0) containing lysozyme (2 µg/mL), 0.15 NaCl, and 2 mM EDTA, then incubated on ice for 30 min and sonicated (five 40-s pulses). The treated cell suspension was incubated for 20 min in a boiling water bath (100 °C) with a periodic stirring, then quickly cooled to 0 °C on ice and centrifuged for 30 min at 4,000 g. PPIase-containing supernatant was filtered through a 10-kDa selective MWCO membrane (Millipore Corp., USA) using an Amicon 8050 cell (Millipore Corp., USA). The obtained concentrated permeate was then freeze-dried.

### 2.2. PPIase purification

Since the C-end of PPIase contains three histidine residues [31], enzyme purification was carried out using a Sepharose-Ni^2+^ column (25 × 50 mm; Pharmacia, Sweden) equilibrated with 50 mM Tris-HCl buffer (pH 7.5) containing 0.25 M NaCl. Freeze-dried enzyme was resuspended in a small volume of a 50 mM Tris-HCl buffer (pH 7.5) containing 1 M NaCl, loaded onto the column, and washed with the same buffer. The enzyme remained on the column due to a binding of its Hys residues with Ni^2+^. Elution was performed by a 300 mL of linear imidazole gradient at a flow rate of 4 mL/min. The eluate was dialyzed at 4 °C against 2 L of distilled water, which pH was adjusted to 7.0 by NaOH, and then freeze-dried. Purity of the obtained protein solution was evaluated by PAGE; a 17-kDa band corresponded to a purified PPIase. For experiments, lyophilized PPIase was dissolved in sterile distilled water up to a concentration of 1 µg/mL.

### 2.3. Obtaining of ALG-based microparticles loaded with PPIase

To produce ALG-based microparticles, the following components were used: 20 mL of 3.3% lecithin solution in heptane (emulsifying agent); 2 mL of ALG solution in water (100 mg or 0.5 mmoles per a link); 0.67 mL of 2.25 М CaCl_2_ (crosslinking agent); 0.5 mL of BSA-PPIase water mix containing 20 mg of the enzyme and 80 mg of bovine serum albumin (BSA).

Water solutions of BSA-PPIase and ALG were mixed and added drop by drop to the emulsifying agent under constant stirring on a high-speed homogenizer (15,000 rpm). Then the whole volume of the crosslinking agent was added to the mix using a syringe. The obtained microparticles were thrice washed with isopropanol, precipitated by centrifugation (1,500 rpm, 5 min), and spray-dried.

According to experimental data, the optimal ALG/PPIase ratio was 9:1. The kinetics of a target protein release from a microparticle depends on the content of the loaded protein and ratio between the fixed protein and free volume of a microparticle. If the free volume is too high, the release rate of target protein decreases. To saturate a free volume of microparticles, we used mix of PPIase and BSA [33],[34]. In addition to ALG-BSA-PPIase microparticles, ALG-BSA microparticles were produced to evaluate a possible eliciting activity of ALG.

Morphology of the obtained ALG complexes was studied using a JEOL JSM-638 OLA scanning electron microscope (FEI Company, USA). Dried microparticles were applied onto an objective table covered by adhesive type, and a palladium layer was sprayed upon the sample.

For experiments, lyophilized microparticles were dissolved in sterile distilled water up to a concentration of 10 µg/mL; in the case of the ALG-BSA-PPIase complex, this concentration corresponded to 1 µg/mL of PPIase.

### 2.4. Plant pathogens

Eliciting activity of the studied compounds was evaluated using three different pathogens, tobacco mosaic virus (TMV), *Alternaria longipes* causing tobacco brown rot, and *Stagonospora nodorum* causing stagonospora nodorum blotch of wheat.

A LS-1 strain of TMV isolated from tobacco plants of the Esheri tobacco plantation (Sukhumi, Georgia) and deposited into the State Collection of Phytopathogenic Microorganisms of the All-Russian Research Institute of Phytopathology (ARRIP) was maintained at the ARRIP Molecular Biology Department on tobacco plants (cv. Samsun). TMV-infected plants were grown in a climatic chamber at a 16-h photoperiod and 24 °C/20 °С (day/night). New plants were inoculated as required by rubbing of their leaves with a TMV suspension representing mix of carborundum and juice of an infected plant [4].

*A. longipes* strain 100055 was provided by the State Collection of Phytopathogenic Microorganisms. The pathogen was grown in sterile Petri plates with potato glucose agar for 2–3 days at 22 °С. Then plates were incubated for 7–8 days in a chamber with UV background illumination. Spore suspension was obtained by washing Petri plates with sterile 50 mM glucose and adjusted to a working concentration of 10^5^ spores/mL.

*S. nodorum* strain В-24/МС2 was provided on Petri plates by the State Collection of Phytopathogenic Microorganisms. The fungus was grown in flasks containing sterile wheat grain (2/3 of a flask volume) supplemented with water (half of the grain weight). Prepared flasks were autoclaved for 1 h (114 °С, 0.5 atm). The resulted grain mass was cooled and inoculated with mycelium collected from the surface of agar medium in obtained Petri plates. Inoculated flasks were incubated for 7 days at 26 °С, then spores were washed down by sterile distilled water and their concentration was adjusted to a working concentration of 10^5^ spores/mL.

### 2.5. Plant cultivation

Tobacco seeds (necrose-forming cv. Xanthi NN) sere sown into vegetation pots filled with soil. Two weeks after shoot appearance, plants were transplanted into individual 400-mL pots and grown in a climatic chamber at a 16-h photoperiod and 22 °C/20 °С (day/night). Plants with 3–4 true leaves were used in experiments. Since leaves from different layers may differently response to infection, we used only mid-layer leaves.

Seeds of wheat (cv. Mironovskaya 808) were washed with water, disinfected for 15 min in 5% KMnO_4_ solution, and placed onto wet filter paper in Petri plates. After 48-h incubation in a thermostat at 22 °С, germinated seeds were sown into vegetation pots filled with soil. Wheat plants were grown in a climatic chamber for 8–10 days at a 16-h photoperiod and 22 °C/20 °С (day/night). A completely unrolled first leaves were used in the study.

### 2.6. Comparison of the eliciting activity of PPIase and ALG-BSA-PPIase complex after treatment with proteinase K

**Figure 1.**
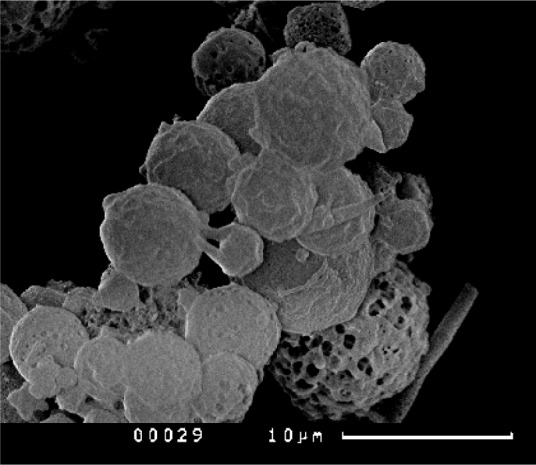
Morphology of ALG-based microparticles loaded with PPIase and BSA.

The experiment was performed using detached tobacco leaves and three different proteinase K concentrations (0, 20, and 100 µg/mL). The left half of each leaf (control) was sprayed, depending on experimental variant, by either distilled water, or proteinase K solution (Table 1). The right half (experiment) was sprayed by PPIase (1 µg/mL) or ALG-BSA-PPIase (10 µg/mL) solutions in the presence of absence of proteinase K (see the corresponding rows in Table 1 and Figure 1 in the Results section). Treated leaves were placed into a wet chamber and incubated 24 h at room temperature. Then the leaves were inoculated with TMV by their rubbing with the mix of carborundum and TMV suspension [4] and left in a wet chamber for five days. TMV suspension used in this and further experiments represented a homogenate of a TMV-infected tobacco leaf diluted by distilled water so that its spraying upon a surface of tobacco leaf provided the development of ∼50 necroses per a leaf half. After 5-day incubation, the number of necroses for each leaf half was counted and the control/experiment ratio of necroses number was calculated for each leaf. The experiment was carried out in four replications.

**Table 1.** Scheme of the experiment on the evaluation of proteinase K effect on eliciting activity of PPIase and ALG-BSA-PPIase complex.

Variants	Proteinase K concentration in the inoculate, µg/mL		
	0	20	100
Control (Proteinase K), µg/mL	0	20	100
Intact PPIase concentration, µg/mL	1	1	1
ALG-BSA-PPIase concentration, µg/mL	10	10	10

### 2.7. Comparison of the eliciting activity of PPIase and ALG-BSA-PPIase complex after UV treatment

PPIase or ALG-BSA-PPIase were dissolved in a 200 µL of sterile distilled water up to a final concentration of 1 and 10 µg/mL, respectively, and poured onto a surface of watch glass. Both glasses were placed into wet chamber and UV-treated using a Mineral Light G-80 UV lamp (36 W, 210–320 nm). The distance between the lamp and preparations was 43 cm. The UV treatment was carried out for 8 h at room temperature; within this period, no significant water evaporation from the solutions was observed. During irradiation, aliquots of both preparations were sampled at a certain time interval to evaluate their eliciting activity in a bioassay using a “tobacco-TMV” model system (see the next subsection). The experiment was carried out in four replications.

### 2.8. Assessment of eliciting activity of the studied preparations using “tobacco-TMV” and “tobacco-A. longipes” model systems

Left halves of detached tobacco leaves were sprayed with PPIase or ALG-BSA-PPIase preparations (1 and 10 µg/mL, respectively), whereas right halves were sprayed with distilled water (control). After 24-h incubation in a wet chamber at room temperature, leaves were inoculated with TMV as described above or sprayed with *A. longipes* spore suspension [9]. After 3–4 days of incubation in a wet chamber, the number of resulted necroses was calculated for each leaf half. Leaf infection level was calculated as a ratio between the number of necroses on the experimental and control leaf halves. The experiment was carried out in five replications.

### 2.9. Assessment of eliciting activity of the studied preparations using a “wheat-S. nodorum” model system

For this model system, the experiment was designed according to the scheme described in [35] and developed for evaluation of resistance of wheat cultivars to *Stagonospora nodorum* with some modifications intended to reveal induced resistance. Unrolled first leaves of wheat plants were detached, and 8-cm fragments were cut out of the middle part of each leaf. Leaf fragments were placed into Petri plates with 1% water agar supplemented with benzimidazole (40 mg/L); the orientation of all leaf fragments was the same in relation to the top and bottom leaf ends. Using a micropipette, 10 µL of the tested preparation (PPIase, ALG-BSA, or ALG-BSA-PPIase) was dropped on the upper part of a leaf fragment, while 10 µL of sterile distilled water was dropped on the bottom part of the same leaf fragment as a control. Petri plates with leaf fragments were incubated for 18 h at a 16-h photoperiod and 22 °С/20 °С (day/night), then all drops of water and tested solutions were removed by filter paper, and spore suspension of *S. nodorum* was dropped on the same places in both top and bottom parts of leaf fragments. After inoculation, Petri plates were first placed into a thermostat for 24 h at 20 °С, then transferred into a climatic chamber and incubated for 7 days at a 16-h photoperiod and 22 °С/20 °С (day/night). At the end of incubation, the level of affection at the treated and control leaf areas was evaluated using the following scale [35]:

0: no symptoms;

1: small dark necroses (1–2 mm in diameter);

2: dark-brown clearly bordered growing spots without chlorosis, leaf tissue remains green;

3: light-brown or brown growing spots edged with chlorosis;

4: light-brown rapidly growing spots without clear borders, pycnid formation is observed.

The level of disease suppression (*E*) was further calculated using the following formula:

$$E=\left(1-\frac{a_{\exp}}{a_{con}}\right)\cdot 100\%,$$

where *a_exp_* and *a_con_* were averaged scores for the experimental and control variants. And the experiment was carried out in 10 replications.

## 3. Results

### 3.1. Production of ALG-based microparticles loaded with PPIase

The development of technology for production of a target biopolymer complex resulted in the obtaining of spherical microparticles ∼5 µm in diameter containing 70% of ALG (carrier), 20% of BSA (inert excipient), and 10% of PPIase as active component (Figure 1).

### 3.2. Comparison of the eliciting activity of PPIase and ALG-BSA-PPIase complex after treatment with proteinase K

**Figure 2.**
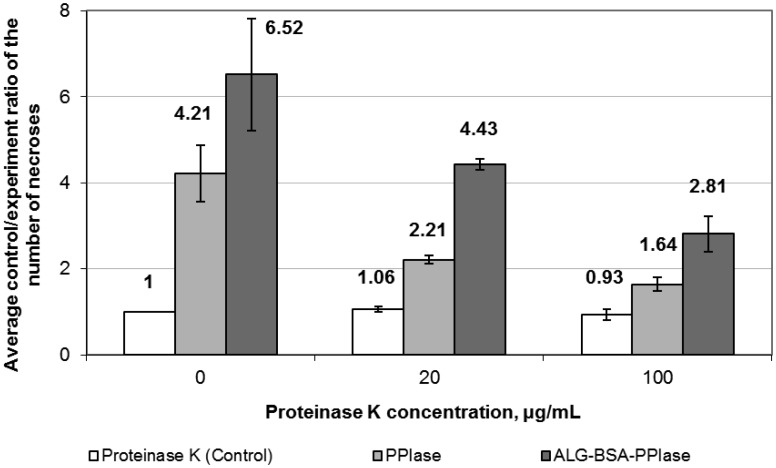
Effect of proteinase K treatment on the eliciting activity of PPIase and ALG-BSA-PPIase complex in a “tobacco-TMV” model system. White bars represent control (treatment with water solution of proteinase K of the corresponding concentration), grey and dark-grey bars represent experimental variants (PPIase or ALG-BSA-PPIase treated with a proteinase K solution of the corresponding concentration.

Results of assessment of eliciting activity of PPIase and ALG-BSA-PPIase treated with proteinase K are shown in Figure 2. Treatment with proteinase K reduced the efficiency of eliciting action of both studied preparations; the higher the proteinase K concentration, the more pronounced the effect. At the same time, the eliciting activity of ALG-BSA-PPIase remained higher than that of PPIase.

### 3.3. Comparison of the eliciting activity of PPIase and ALG-BSA-PPIase complex after UV treatment

**Figure 3.**
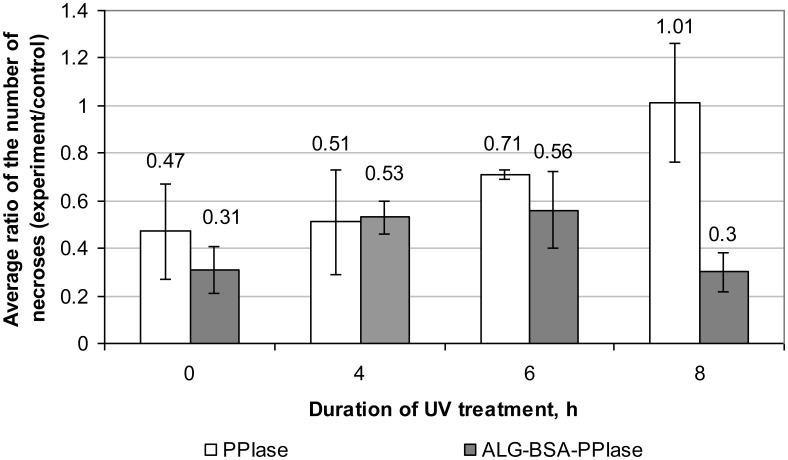
Effect of UV treatment on the eliciting activity of PPIase and ALG-BSA-PPIase complex in a “tobacco-TMV” model system. Control was represented by left leaf halves treated with sterile distilled water; while experiment variants were right leaf halves treated with one of the tested preparations.

Results of assessment of eliciting activity of UV-treated PPIase and ALG-BSA-PPIase in relation to the “tobacco-TMV” model system are shown in Figure 3. Within 6 h of treatment, the eliciting activity of both preparations was about the same. A 8-h UV irradiation caused a significant reduction of the PPIase activity, whereas that of ALG-BSA-PPIase remained at the same level. Therefore, ALG provides some UV protection for PPIase.

**Figure 4.**
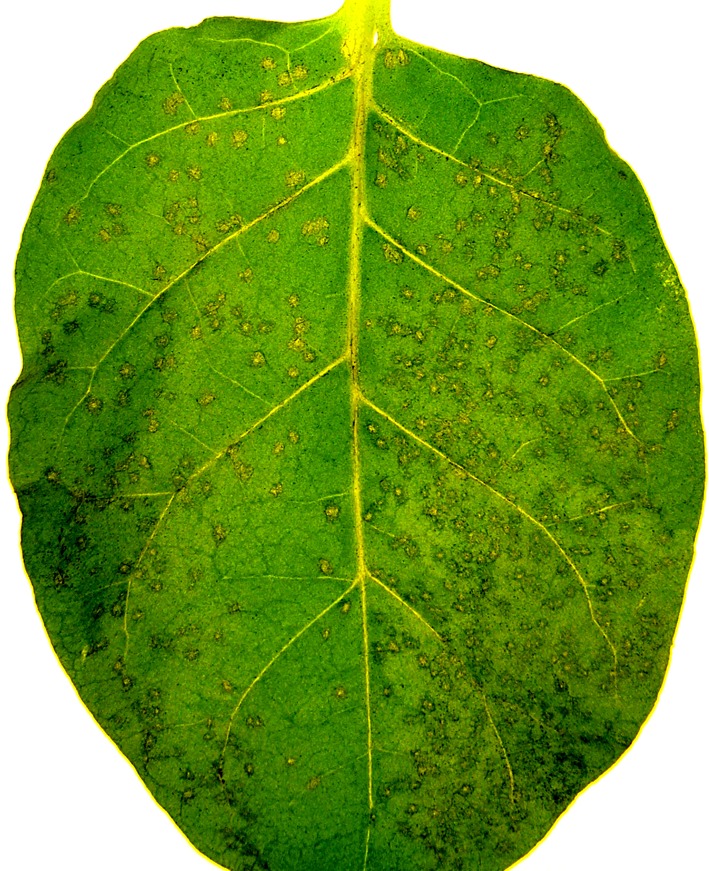
Tobacco leaf treated with PPIase (right half) and ALG-BSA-PPIase (lef half) preparations preliminarily undergone to a 8-h UV irradiation.

At the next stage, experimental design was changed so that two halves of the same leaf were separately sprayed with PPIase (control) and ALG-BSA-PPIase, which were preliminarily treated with UV for 8 h. Results of this experiment are shown in Table 2. A typical appearance of treated leaves is shown in Figure 4.

**Table 2.** Number of necroses on the halves of tobacco leaves sprayed with UV-treated PPIase and ALG-BSA-PPIase preparations (*p* < 0.01).

Preparation	Number of necroses				Average value, М ± SE
	Leaf 1	Leaf 2	Leaf 3	Leaf 4	
ALG-BSA-PPIase (Experiment)	79	41	63	53	59.0 ± 8.0
PPIase (Control)	127	85	94	139	111.3 ± 12.9
Experiment/Control ratio	0.62	0.48	0.67	0.38	0.54 ± 0.06

According to the obtained data, in the case of prolonged UV irradiation, ALG-BSA-PPIase provides twice higher eliciting activity than PPIase alone. In the further studies, similar experiments will be carried out for the “tobacco-*A. longipes*” model system.

### 3.4. Comparison of the eliciting activity of PPIase, ALG-BSA, and ALG-BSA-PPIase complex in a “tobacco-TMV” model system

Results of assessment of the eliciting activity of PPIase, ALG-BSA, and ALG-BSA-PPIase in relation to the “tobacco-TMV” model system are shown in Table 3. According to these data, no significant difference was revealed between PPIase and ALG-BSA-PPIase; in both cases, the average amount of necroses per a leaf decreased in 32–35 times as compared with the control. In the case of ALG-BSA, no eliciting activity was revealed for the concentration used.

**Table 3.** Level of affection of tobacco leaves with tobacco mosaic virus (TMV) after their treatment with PPIase and ALG-based complexes.

Preparations	Average number of necroses	
	leaf halves treated with the preparation	Leaf halves treated with sterile distilled water (control)
ALG-BSA-PPIase	1.0 ± 0.3^а^	34.8 ± 7.6^b^
PPIase	1.0 ± 0.4^а^	32.2 ± 4.7^b^
ALG-BSA	22.2 ± 4.7^b^	18.5 ± 4.1^b^

Note: Difference between the values indicated with different letters is significant at *р* < 0.05.

### 3.5. Comparison of the eliciting activity of PPIase, ALG-BSA, and ALG-BSA-PPIase complex in a “tobacco-A. longipes” model system

**Figure 5.**
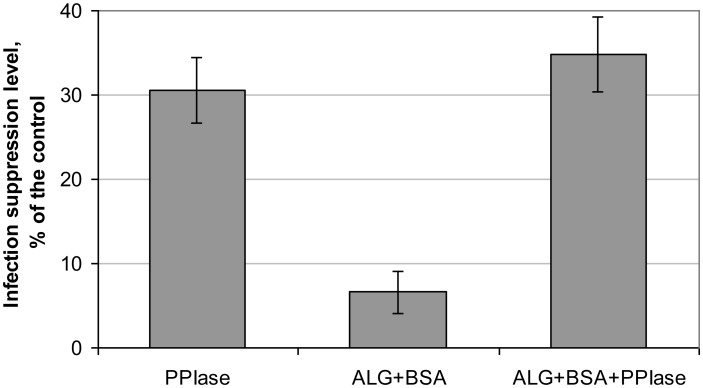
Effect of treatment of tobacco leaves with PPIase and ALG-based complexes on the level of suppression of the development of brown rot of tobacco caused by *Alternaria longipes*.

Results of assessment of the eliciting activity of PPIase, ALG-BSA, and ALG-BSA-PPIase in relation to the “tobacco-*A. longipes*” model system are shown in Figure 5. For both PPIase and ALG-BSA-PPIase, infection suppression level was about the same (30.5 and 34.8%, respectively), i.e., complexation did not influence on the eliciting properties of PPIase in this model system. ALG-BSA complex also demonstrated a small eliciting effect (6.6%) in relation to the target pathogen.

### 3.6. Comparison of the protective activity of PPIase, ALG-BSA, and ALG-BSA-PPIase complex in a “wheat-S. nodorum” model system

**Figure 6.**
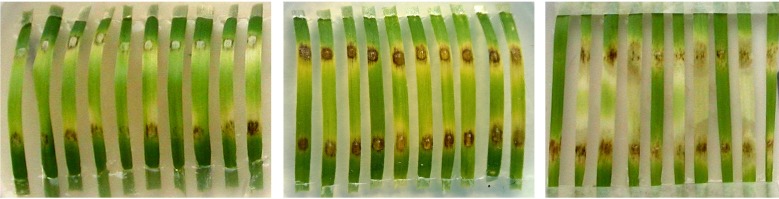
Effect of induced resistance in wheat against *Stagonospora nodorum* caused by treatment with PPIase and ALG-BSA-PPIase. Left: drops of ALG-BSA-PPIase and sterile distilled water (control) were placed on the upper and bottom part of leaves, respectively, and after 24-h incubation were removed and replaced by drops of *S. nodorum* spore suspension. Center: drops of *S. nodorum* spore suspension were placed on the top and bottom part of leaves. Right: drops of PPIase and sterile distilled water (control) were placed on the upper and bottom part of leaves, respectively, and after 24-h incubation were removed and replaced by drops of *S. nodorum* spore suspension.

**Figure 7.**
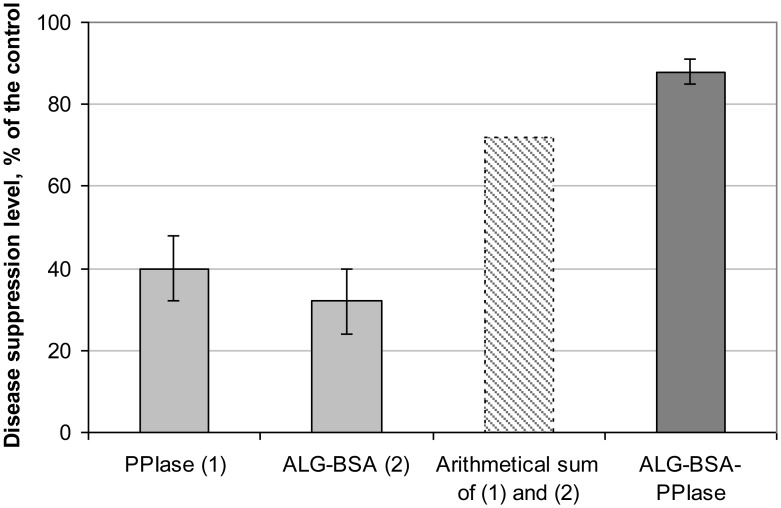
Effect of treatment of wheat leaves with PPIase, ALG-BSA, and ALG-BSA-PPIase preparations on the suppression of the *Septoria nodorum* blotch disease caused by *Septoria nodorum*. Hatched bar shows expected arithmetical sum of suppression effects provided by PPIase and ALG.

Results of assessment of the eliciting activity of PPIase, ALG-BSA, and ALG-BSA-PPIase in relation to the “wheat-*S. nodorum*” model system are shown in Figures 6 and 7. A significant suppression of a disease development by ALG-BSA complex (32.2%) was observed, while BSA alone did not differ from the control, i.e., did not possess such activity. In the case of PPIase, the value of this index was 39.8%. The protective effect of ALG-BSA-PPIase (88.4%) was reliably higher than those of other assessed variants and obviously exceeded the sum of the effects provided by ALG-BSA and PPIase alone that indicated a possible synergistic interaction between ALG and PPIase (Figure 7).

To check a hypothesis about synergistic interaction between ALG and PPIase, we used Limpel's formula [36]:

$$E_{e}=X+Y-\left(\frac{XY}{100}\right)$$

where *Х* and *Y* represent effects provided by these two factors separately, and *E_е_* is their expected summarized effect in the case of an additive character of their interaction. If the value of the experimental summarized effect (*E_real_*) exceeds *E_е_*, interaction of these factors is considered to be synergistic.

Results of the corresponding calculations for the “wheat-*S. nodorum*” model system are shown in Table 4. According to the obtained result, *E_е_* = 59.2%, whereas *E_real_* = 88.4% that confirms a synergistic character of the ALG and PPIase eliciting activity.

**Table 4.** Parameters of the Limpel's formula calculated for the effect of ALG and PPIase on the “wheat-*S. nodorum*” model system.

Treatment variant	PPIase (*Х*)	ALG + BSA (*Y*)	Arithmetical sum, *X* + *Y*	Calculated *Е_е_*, %	ALG-BSA-PPIase (*E_real_*)
Disease suppression level, %	39.8	32.2	72.2	59.2	88.4

## 4. Conclusions

The main purpose of this study was the examination of a principal possibility to provide sufficient elicitor activity of a microbial enzyme PPIase under action of adverse environmental factors via its encapsulation in sodium alginate (ALG). A choice of this biopolymer was determined by two factors. First, this compound is widely used in medicine for encapsulation of various therapeutic drugs and their safe delivery to target organs and tissues [37]. Relatively soft conditions of alginate hydrogel formation provide a possibility to use it as a carrier of proteins, DNA, and live cells while keeping their biological activity unchanged; a choice of a certain type of this biopolymer allows one to regulate pore size, rate of degradation of the formed biopolymeric complexes, and release rate of a target compound [38]. Second, ALG itself is able to stimulate growth and development of plants and possesses some eliciting activity [39]. For example, this compound induces defense response in grape cells via the enhancement of transcription of genes encoding chitinase and gluconase [24] and improves TMV resistance of tobacco plants [40].

In the course of this study, we first encapsulated PPIase via its immobilization on ALG microparticles loaded with BSA. As a result, elicitor activity of the enzyme remained at the same level even after a 8-h UV treatment, whereas the same treatment of PPIase alone caused loss of its effect towards TMV. These results agree with the existing data on the ALG ability to provide UV protection of some bioactive compounds in the case of their complexation [41],[42]. In addition, PPIase encapsulation provided partial protection of the enzyme from proteinase K that also corresponds to the data on the use of ALG for protection of protein- or peptide-based drugs from acids and degrading enzymes of a gastrointestinal tract [43].

A comparison of the eliciting activity of free and encapsulated PPIase performed in “tobacco-TMV” and “tobacco-*A. longipes*” model system showed lack of any negative effect of the encapsulation process on the PPIase ability to stimulate suppression of these two pathogens by plant immune system. The obtained data also allowed us to conclude that the ALG-BSA complex has some eliciting activity in relation to suppression of *A. longipes* development on tobacco plants that agrees with the existing data on the eliciting activity of ALG [24],[40]. At the same time, we did not observe such ALG activity in relation to TMV that contradicts data published by Laporte et al. [40]. According to these authors, tobacco treatment with water solution of algal polysaccharides may improve TMV resistance of tobacco plants by 74%. Such inconsistency of data may be probably explained by a significant difference in the experimental design: authors of the above-mentioned study treated tobacco plants with ALG for one month on a weekly basis, and examined TMV resistance 7 and 15 days after the last treatment. In our further studies we plan to examine eliciting abilities of the studied preparations for other plant treatment schemes.

A comparison of the eliciting activity of the studied preparations performed in a “wheat- *S. nodorum*” model system showed that the development of a target plant pathogen on wheat leaf fragments treated with encapsulated PPIase was almost twice lower as compared to free PPIase. In addition, we observed a significant eliciting activity of ALG-BSA complex within this model system and also revealed a synergistic character of PPIase and ALG stimulation of wheat resistance to *S. nodorum*. As far as we know, this is the first observation of a synergistic interaction between ALG and other compound possessing bioactive (eliciting) properties. The further detailed study of this phenomenon is required.

Results of this study confirmed a principal possibility to develop environmentally safe biopesticides based on the biopolymeric complex of PPIase and ALG and characterized by improved resistance of the bioactive component to adverse biotic and abiotic factors and significant eliciting activity in relation to a range of taxonomically distant plant pathogens. As we have already mentioned, a possibility to use ALG for production of encapsulated microbial biopreparations for soil fertilization has been reported in a series of studies [29],[30]. One of the recently published studies was devoted to the development of ALG microcapsules containing plant growth stimulator based on a *Bacillus subtilis* strain [44]; according to authors, such encapsulation of bacterial cells significantly improved their survival in soil and provided a possibility of commercial production of such preparations. In our case, a wide range of action of PPIase and its high heat resistance facilitating the isolation and purification of this protein [9] represent additional advantages for the industrial production of PPIase-based biopesticides. In our future work, we plan to continue the study of properties of encapsulated PPIase using other model systems and whole plants.
